# A model of evolution with constant selective pressure for regulatory DNA sites

**DOI:** 10.1186/1471-2148-7-125

**Published:** 2007-07-27

**Authors:** Farida N Enikeeva, Ekaterina A Kotelnikova, Mikhail S Gelfand, Vsevolod J Makeev

**Affiliations:** 1 Institute for Information Transmission Problems (the Kharkevich Institute) of RAS, Bolshoi Karetny pereulok, 19, GSP-4, Moscow, 127994, Russia; 2 State Research Institute of Genetics and Selection of Industrial Microorganisms, 1st Dorozhnyj proezd, 1, Moscow, 113535, Russia; 3 Faculty of Bioengineering and Bioinformatics, Moscow State University, Vorobyevy Gory 1-73, Moscow, 119992, Russia; 4 Ariadne Genomics Inc. 9700 Great Seneca Highway, Suite 113, Rockville, MD 20850, USA; 5 Engelgardt Institute of Molecular Biology of RAS, Vavilova 32, Moscow, 119991, Russia

## Abstract

**Background:**

Molecular evolution is usually described assuming a neutral or weakly non-neutral substitution model. Recently, new data have become available on evolution of sequence regions under a selective pressure, e.g. transcription factor binding sites. To reconstruct the evolutionary history of such sequences, one needs evolutionary models that take into account a substantial constant selective pressure.

**Results:**

We present a simple evolutionary model with a single preferred (consensus) nucleotide and the neutral substitution model adopted for all other nucleotides. This evolutionary model has a rate matrix in which all substitutions that do not involve the consensus nucleotide occur with the same rate. The model has two time scales for achieving a stationary distribution; in the general case only one of the two rate parameters can be evaluated from the stationary distribution. In the middle-time zone, a counterintuitive behavior was observed for some parameter values, with a probability of conservation for a non-consensus nucleotide greater than that for the consensus nucleotide. Such an effect can be observed only in the case of weak preference for the consensus nucleotide, when the probability to observe the consensus nucleotide in the stationary distribution is less than 1/2. If the substitution rate is represented as a product of mutation and fixation, only the fixation can be calculated from the stationary distribution. The exhibited conservation of non-consensus nucleotides does not take place if the elements of mutation matrix are identical, and can be related to the reduced mutation rate between the non-consensus nucleotides. This bias can have no effect on the stationary distribution of nucleotide frequencies calculated over the ensemble of multiple alignments, e.g. transcription factor binding sites upstream of different sets of co-regulated orthologous genes.

**Conclusion:**

The derived model can be used as a null model when analyzing the evolution of orthologous transcription factor binding sites. In particular, our findings show that a nucleotide preferred at some position of a multiple alignment of binding sites for some transcription factor in the same genome is not necessarily the most conserved nucleotide in an alignment of orthologous sites from different species. However, this effect can take place only in the case of a mutation matrix whose elements are not identical.

## Background

The controlled expression of genes is the main mechanism responsible for the life cycle and biodiversity [1]. Transcription, which is a crucial process defining the level of gene expression, is regulated by interaction of transcription factor proteins (TFs) with transcription factor binding sites (TFBSs) in a DNA molecule [2]. Thus, adaptable interaction between TFs and TFBSs is one of the main driving forces of biological evolution [3].

Both TFs and TFBSs are subject to mutations and selection affecting their interaction [4]. In this study we focus on mutations in TFBSs. New experimental [5-7] and computational [8] methods of TFBS identification produce an increasing amount of data about TFBS sequences, which creates a possibility to study evolutionary events in these regions.

Modelling evolution of regulatory sequences can be useful for understanding both the general mechanisms of gene expression control and the regulatory history of particular genes.

The evolution of regulatory regions has a complex pattern [3,9-11] and is still unclear in many aspects. DNA segments can gain and lose the TFBS function [12], and that can bring new genes under regulation by a particular TF [13-15], or divert regulation from other genes [16,17]. One particular type of events is emergence of new or changed sites following displacement of a transcription factor by horizontal transfer [18]. Sometimes this leads to considerable changes in the regulon content [19,20] or even partial or complete rewiring of regulatory cascades [21-26], reviewed in [27].

Evolution of functional TFBS sequences is strongly non-neutral [11,28] and under a positive selection [29], which makes it difficult to calculate the rate of TFBS evolution. This rate varies between TFBS positions [30,31]. Moreover, co-evolution of TFBS and TF [16,20] can make the selective pressure vary in different lineages. Indeed, although in some cases the DNA motif bound by orthologous factors may be conserved at surprisingly large evolutionary distances [14,32-34], in other cases not only the motifs themselves may be different [35,36], but even the symmetry of the motif (e.g. palindrome or direct repeat) may change [37,38].

The existing evolutionary models, which were successful in reconstruction of phylogenetic relations, can be applied to evolution of regulatory sequences only with a caution. Such models are historically related to the Jukes–Cantor [39] and Kimura [40] models of molecular evolution. Existing modifications of these models take into account various global characteristics like transition/transversion rate or local GC composition [41-44]. They are not applicable to the case of strong selection for a specific nucleotide at a particular position.

On the other hand, models developed specifically for the evolution of TFBS are needed to reconstruct the evolutionary origin of a particular TFBS and to evaluate the position-specific mutation rate and selective pressure.

Because of the position-specific variation in the rate of TFBS evolution [30], the rate matrix must also be position-specific. The data produced by mass experiments on TFBS identification or comparative genomic studies produce tens of TFBS for each TF (more exactly, a group of orthologous TFs). That might be sufficient to evaluate the evolutionary rate at each TFBS position.

Here we consider the simplest model of position-specific evolution with one preferred (consensus) nucleotide and three other (minor) nucleotides, the latter considered in a symmetric setting, without any selection or rate preferences [45]. Such a model can be deduced from physical requirements of the TF/TFBS interaction [46] and can explain the observed TFBS fuzziness.

We build a rate matrix, which enhances the model of [45]. We calculate the substitution probability for each finite time and show that the nucleotide conservation in phylogenetic lineages can be non-trivial for some parameter values. Particularly, a non-consensus nucleotide may appear more conserved than the consensus nucleotide, although the latter has a selective preference. This happens when the rate of mutations between non-consensus nucleotides is lower than the rate of mutation into the consensus, or there is selection against any mutation in non-consensus nucleotides.

## Results and Discussion

### Model

We start with definitions. We consider an alignment of several sequences; all positions in this alignment are assumed to be independent, and thus may be modelled independently. *Consensus nucleotide *(or simply *consensus*) is the most frequent nucleotide in an alignment column (*position*). Other nucleotides are called *non-consensus*. The frequency of the consensus nucleotide *N*_*c *_is a fraction of the number of consensus nucleotides in a particular position. Obviously, 1/4 <*N*_*c *_≤ 1. The consensus is called *weak*, if 1/4 <*N_c _*< 1/2; the consensus is *strong*, if 1/2 ≤ *N_c _*< 1.

Consider the model of nucleotide substitutions given by a Markov process *X*(*t*) with four states {*g*_1_, *g*_2_, *g*_3_, *g*_4_}. Without loss of generality, assume that the state *g*_1 _is the consensus state and the states *g*_2_, *g*_3_, *g*_4 _are equiprobable non-consensus states. Suppose that the transition rate matrix *A *= (*q*_*ij*_) is given by

A=(−3ααααβ−β−2δδδβδ−β−2δδβδδ−β−2δ),
 MathType@MTEF@5@5@+=feaafiart1ev1aaatCvAUfKttLearuWrP9MDH5MBPbIqV92AaeXatLxBI9gBaebbnrfifHhDYfgasaacH8akY=wiFfYdH8Gipec8Eeeu0xXdbba9frFj0=OqFfea0dXdd9vqai=hGuQ8kuc9pgc9s8qqaq=dirpe0xb9q8qiLsFr0=vr0=vr0dc8meaabaqaciaacaGaaeqabaqabeGadaaakeaacqWGbbqqcqGH9aqpdaqadaqaauaabeqaeqaaaaaabaGaeyOeI0IaeG4mamdcciGae8xSdegabaGae8xSdegabaGae8xSdegabaGae8xSdegabaGae8NSdigabaGaeyOeI0Iae8NSdiMaeyOeI0IaeGOmaiJae8hTdqgabaGae8hTdqgabaGae8hTdqgabaGae8NSdigabaGae8hTdqgabaGaeyOeI0Iae8NSdiMaeyOeI0IaeGOmaiJae8hTdqgabaGae8hTdqgabaGae8NSdigabaGae8hTdqgabaGae8hTdqgabaGaeyOeI0Iae8NSdiMaeyOeI0IaeGOmaiJae8hTdqgaaaGaayjkaiaawMcaaiabcYcaSaaa@5A47@

where *q*_*ij *_is the transition rate from the state *g*_*i *_to *g*_*j *_and *α*, *β*, and *δ *are positive unknown parameters.

### Transitional probabilities

Let **P**_*ij*_(*t*) be the transitional probability from the state *g*_*i *_to the state *g*_*j *_for the time *t*. From the theory of Markov chains we have for *h *→ 0

1−Pii(h)=−qiih+o(1),Pij(h)=qijh+o(1),i≠j
 MathType@MTEF@5@5@+=feaafiart1ev1aaatCvAUfKttLearuWrP9MDH5MBPbIqV92AaeXatLxBI9gBaebbnrfifHhDYfgasaacH8akY=wiFfYdH8Gipec8Eeeu0xXdbba9frFj0=OqFfea0dXdd9vqai=hGuQ8kuc9pgc9s8qqaq=dirpe0xb9q8qiLsFr0=vr0=vr0dc8meaabaqaciaacaGaaeqabaqabeGadaaakeaafaqaceGaeaaaaeaacqaIXaqmcqGHsislieqacqWFqbaudaWgaaWcbaGaemyAaKMaemyAaKgabeaakiabcIcaOiabdIgaOjabcMcaPaqaaiabg2da9aqaaiabgkHiTiabdghaXnaaBaaaleaacqWGPbqAcqWGPbqAaeqaaOGaemiAaGMaey4kaSIaem4Ba8MaeiikaGIaeGymaeJaeiykaKIaeiilaWcabaaabaGae8huaa1aaSbaaSqaaiabdMgaPjabdQgaQbqabaGccqGGOaakcqWGObaAcqGGPaqkaeaacqGH9aqpaeaacqWGXbqCdaWgaaWcbaGaemyAaKMaemOAaOgabeaakiabdIgaOjabgUcaRiabd+gaVjabcIcaOiabigdaXiabcMcaPiabcYcaSaqaaiabdMgaPjabgcMi5kabdQgaQbaaaaa@5B54@

For brevity we denote by 'c' the subscript '1' corresponding to the consensus state *g*_1 _and by 'n' and 'm' the subscripts 2, 3, and 4 corresponding to the non-consensus states *g*_2_, *g*_3_, *g*_4_. Thus we have five transitional probabilities **P**_*cc*_, **P**_*cn*_, **P**_*nc*_, **P**_*nn*_, and **P**_*nm*_, where *n *and *m *stand for two distinct non-consensus states. The formulas for **P**_*ij *_are derived from simple calculation:

Pcc(t)=β3α+β+3α3α+βe−(3α+β)t,Pcn(t)=α3α+β−α3α+βe−(3α+β)t,Pnc(t)=β3α+β−β3α+βe−(3α+β)t,Pnn(t)=α3α+β+β3(3α+β)e−(3α+β)t+23e−(3δ+β)t,Pnm(t)=α3α+β+β3(3α+β)e−(3α+β)t−13e−(3δ+β)t.
 MathType@MTEF@5@5@+=feaafiart1ev1aaatCvAUfKttLearuWrP9MDH5MBPbIqV92AaeXatLxBI9gBaebbnrfifHhDYfgasaacH8akY=wiFfYdH8Gipec8Eeeu0xXdbba9frFj0=OqFfea0dXdd9vqai=hGuQ8kuc9pgc9s8qqaq=dirpe0xb9q8qiLsFr0=vr0=vr0dc8meaabaqaciaacaGaaeqabaqabeGadaaakeaafaqaaeqbdaaaaeaaieqacqWFqbaudaWgaaWcbaGaem4yamMaem4yamgabeaakiabcIcaOiabdsha0jabcMcaPaqaaiabg2da9aqaamaalaaabaacciGae4NSdigabaGaeG4mamJae4xSdeMaey4kaSIae4NSdigaaiabgUcaRmaalaaabaGaeG4mamJae4xSdegabaGaeG4mamJae4xSdeMaey4kaSIae4NSdigaaiabdwgaLnaaCaaaleqabaGaeyOeI0IaeiikaGIaeG4mamJae4xSdeMaey4kaSIae4NSdiMaeiykaKIaemiDaqhaaOGaeiilaWcabaGae8huaa1aaSbaaSqaaiabdogaJjabd6gaUbqabaGccqGGOaakcqWG0baDcqGGPaqkaeaacqGH9aqpaeaadaWcaaqaaiab+f7aHbqaaiabiodaZiab+f7aHjabgUcaRiab+j7aIbaacqGHsisldaWcaaqaaiab+f7aHbqaaiabiodaZiab+f7aHjabgUcaRiab+j7aIbaacqWGLbqzdaahaaWcbeqaaiabgkHiTiabcIcaOiabiodaZiab+f7aHjabgUcaRiab+j7aIjabcMcaPiabdsha0baakiabcYcaSaqaaiab=bfaqnaaBaaaleaacqWGUbGBcqWGJbWyaeqaaOGaeiikaGIaemiDaqNaeiykaKcabaGaeyypa0dabaWaaSaaaeaacqGFYoGyaeaacqaIZaWmcqGFXoqycqGHRaWkcqGFYoGyaaGaeyOeI0YaaSaaaeaacqGFYoGyaeaacqaIZaWmcqGFXoqycqGHRaWkcqGFYoGyaaGaemyzau2aaWbaaSqabeaacqGHsislcqGGOaakcqaIZaWmcqGFXoqycqGHRaWkcqGFYoGycqGGPaqkcqWG0baDaaGccqGGSaalaeaacqWFqbaudaWgaaWcbaGaemOBa4MaemOBa4gabeaakiabcIcaOiabdsha0jabcMcaPaqaaiabg2da9aqaamaalaaabaGae4xSdegabaGaeG4mamJae4xSdeMaey4kaSIae4NSdigaaiabgUcaRmaalaaabaGae4NSdigabaGaeG4mamJaeiikaGIaeG4mamJae4xSdeMaey4kaSIae4NSdiMaeiykaKcaaiabdwgaLnaaCaaaleqabaGaeyOeI0IaeiikaGIaeG4mamJae4xSdeMaey4kaSIae4NSdiMaeiykaKIaemiDaqhaaOGaey4kaSYaaSaaaeaacqaIYaGmaeaacqaIZaWmaaGaemyzau2aaWbaaSqabeaacqGHsislcqGGOaakcqaIZaWmcqGF0oazcqGHRaWkcqGFYoGycqGGPaqkcqWG0baDaaGccqGGSaalaeaacqWFqbaudaWgaaWcbaGaemOBa4MaemyBa0gabeaakiabcIcaOiabdsha0jabcMcaPaqaaiabg2da9aqaamaalaaabaGae4xSdegabaGaeG4mamJae4xSdeMaey4kaSIae4NSdigaaiabgUcaRmaalaaabaGae4NSdigabaGaeG4mamJaeiikaGIaeG4mamJae4xSdeMaey4kaSIae4NSdiMaeiykaKcaaiabdwgaLnaaCaaaleqabaGaeyOeI0IaeiikaGIaeG4mamJae4xSdeMaey4kaSIae4NSdiMaeiykaKIaemiDaqhaaOGaeyOeI0YaaSaaaeaacqaIXaqmaeaacqaIZaWmaaGaemyzau2aaWbaaSqabeaacqGHsislcqGGOaakcqaIZaWmcqGF0oazcqGHRaWkcqGFYoGycqGGPaqkcqWG0baDaaGccqGGUaGlaaaaaa@F879@

For simplicity, introduce a new time-scale, *u *= *tβ*, and denote *λ *= *α*/*β*, *μ *= *δ*/*β*. Then

Pcc(u)=13λ+1+3λ3λ+1e−(3λ+1)u,Pcn(u)=λ3λ+1−λ3λ+1e−(3λ+1)u,Pnc(u)=13λ+1−13λ+1e−(3λ+1)u,Pnn(u)=λ3λ+1+13(3λ+1)e−(3λ+1)u+23e−(3μ+1)u,Pnm(u)=λ3λ+1+13(3λ+1)e−(3λ+1)u−13e−(3μ+1)u
 MathType@MTEF@5@5@+=feaafiart1ev1aaatCvAUfKttLearuWrP9MDH5MBPbIqV92AaeXatLxBI9gBaebbnrfifHhDYfgasaacH8akY=wiFfYdH8Gipec8Eeeu0xXdbba9frFj0=OqFfea0dXdd9vqai=hGuQ8kuc9pgc9s8qqaq=dirpe0xb9q8qiLsFr0=vr0=vr0dc8meaabaqaciaacaGaaeqabaqabeGadaaakeaafaqaaeqbdaaaaeaaieqacqWFqbaudaWgaaWcbaGaem4yamMaem4yamgabeaakiabcIcaOiabdwha1jabcMcaPaqaaiabg2da9aqaamaalaaabaGaeGymaedabaGaeG4mamdcciGae43UdWMaey4kaSIaeGymaedaaiabgUcaRmaalaaabaGaeG4mamJae43UdWgabaGaeG4mamJae43UdWMaey4kaSIaeGymaedaaiabdwgaLnaaCaaaleqabaGaeyOeI0IaeiikaGIaeG4mamJae43UdWMaey4kaSIaeGymaeJaeiykaKIaemyDauhaaOGaeiilaWcabaGae8huaa1aaSbaaSqaaiabdogaJjabd6gaUbqabaGccqGGOaakcqWG1bqDcqGGPaqkaeaacqGH9aqpaeaadaWcaaqaaiab+T7aSbqaaiabiodaZiab+T7aSjabgUcaRiabigdaXaaacqGHsisldaWcaaqaaiab+T7aSbqaaiabiodaZiab+T7aSjabgUcaRiabigdaXaaacqWGLbqzdaahaaWcbeqaaiabgkHiTiabcIcaOiabiodaZiab+T7aSjabgUcaRiabigdaXiabcMcaPiabdwha1baakiabcYcaSaqaaiab=bfaqnaaBaaaleaacqWGUbGBcqWGJbWyaeqaaOGaeiikaGIaemyDauNaeiykaKcabaGaeyypa0dabaWaaSaaaeaacqaIXaqmaeaacqaIZaWmcqGF7oaBcqGHRaWkcqaIXaqmaaGaeyOeI0YaaSaaaeaacqaIXaqmaeaacqaIZaWmcqGF7oaBcqGHRaWkcqaIXaqmaaGaemyzau2aaWbaaSqabeaacqGHsislcqGGOaakcqaIZaWmcqGF7oaBcqGHRaWkcqaIXaqmcqGGPaqkcqWG1bqDaaGccqGGSaalaeaacqWFqbaudaWgaaWcbaGaemOBa4MaemOBa4gabeaakiabcIcaOiabdwha1jabcMcaPaqaaiabg2da9aqaamaalaaabaGae43UdWgabaGaeG4mamJae43UdWMaey4kaSIaeGymaedaaiabgUcaRmaalaaabaGaeGymaedabaGaeG4mamJaeiikaGIaeG4mamJae43UdWMaey4kaSIaeGymaeJaeiykaKcaaiabdwgaLnaaCaaaleqabaGaeyOeI0IaeiikaGIaeG4mamJae43UdWMaey4kaSIaeGymaeJaeiykaKIaemyDauhaaOGaey4kaSYaaSaaaeaacqaIYaGmaeaacqaIZaWmaaGaemyzau2aaWbaaSqabeaacqGHsislcqGGOaakcqaIZaWmcqGF8oqBcqGHRaWkcqaIXaqmcqGGPaqkcqWG1bqDaaGccqGGSaalaeaacqWFqbaudaWgaaWcbaGaemOBa4MaemyBa0gabeaakiabcIcaOiabdwha1jabcMcaPaqaaiabg2da9aqaamaalaaabaGae43UdWgabaGaeG4mamJae43UdWMaey4kaSIaeGymaedaaiabgUcaRmaalaaabaGaeGymaedabaGaeG4mamJaeiikaGIaeG4mamJae43UdWMaey4kaSIaeGymaeJaeiykaKcaaiabdwgaLnaaCaaaleqabaGaeyOeI0IaeiikaGIaeG4mamJae43UdWMaey4kaSIaeGymaeJaeiykaKIaemyDauhaaOGaeyOeI0YaaSaaaeaacqaIXaqmaeaacqaIZaWmaaGaemyzau2aaWbaaSqabeaacqGHsislcqGGOaakcqaIZaWmcqGF8oqBcqGHRaWkcqaIXaqmcqGGPaqkcqWG1bqDaaaaaaaa@EAB7@

and

A=β⋅(−3λλλλ1−1−2μμμ1μ−1−2μμ1μμ−1−2μ).
 MathType@MTEF@5@5@+=feaafiart1ev1aaatCvAUfKttLearuWrP9MDH5MBPbIqV92AaeXatLxBI9gBaebbnrfifHhDYfgasaacH8akY=wiFfYdH8Gipec8Eeeu0xXdbba9frFj0=OqFfea0dXdd9vqai=hGuQ8kuc9pgc9s8qqaq=dirpe0xb9q8qiLsFr0=vr0=vr0dc8meaabaqaciaacaGaaeqabaqabeGadaaakeaacqWGbbqqcqGH9aqpiiGacqWFYoGycqGHflY1daqadaqaauaabeqaeqaaaaaabaGaeyOeI0IaeG4mamJae83UdWgabaGae83UdWgabaGae83UdWgabaGae83UdWgabaGaeGymaedabaGaeyOeI0IaeGymaeJaeyOeI0IaeGOmaiJae8hVd0gabaGae8hVd0gabaGae8hVd0gabaGaeGymaedabaGae8hVd0gabaGaeyOeI0IaeGymaeJaeyOeI0IaeGOmaiJae8hVd0gabaGae8hVd0gabaGaeGymaedabaGae8hVd0gabaGae8hVd0gabaGaeyOeI0IaeGymaeJaeyOeI0IaeGOmaiJae8hVd0gaaaGaayjkaiaawMcaaiabc6caUaaa@5B16@

Note that the parameter *μ *is related to the conservation within the set of non-consensus states {*g*_2_, *g*_3_, *g*_4_}. Simply, *λ *is a rate of transition from the consensus nucleotide and *μ *is a rate of transition between non-consensus states up to the time-scale parameter *β*.

The stationary distribution *π *= (*π*_*c*_, *π*_*n*_, *π*_*n*_, *π*_*n*_) of the process *X*(*t*) is given by

π=(13λ+1,λ3λ+1,λ3λ+1,λ3λ+1).
 MathType@MTEF@5@5@+=feaafiart1ev1aaatCvAUfKttLearuWrP9MDH5MBPbIqV92AaeXatLxBI9gBaebbnrfifHhDYfgasaacH8akY=wiFfYdH8Gipec8Eeeu0xXdbba9frFj0=OqFfea0dXdd9vqai=hGuQ8kuc9pgc9s8qqaq=dirpe0xb9q8qiLsFr0=vr0=vr0dc8meaabaqaciaacaGaaeqabaqabeGadaaakeaaiiGacqWFapaCcqGH9aqpdaqadaqaamaalaaabaGaeGymaedabaGaeG4mamJae83UdWMaey4kaSIaeGymaedaaiabcYcaSmaalaaabaGae83UdWgabaGaeG4mamJae83UdWMaey4kaSIaeGymaedaaiabcYcaSmaalaaabaGae83UdWgabaGaeG4mamJae83UdWMaey4kaSIaeGymaedaaiabcYcaSmaalaaabaGae83UdWgabaGaeG4mamJae83UdWMaey4kaSIaeGymaedaaaGaayjkaiaawMcaaiabc6caUaaa@4C94@

According to our definition of the consensus as the most frequent nucleotide, from *π*_*c *_= 1/(3*λ *+ 1) > 1/4 it follows that 0 <*λ *< 1. The parameter *λ *is responsible for transitions from the consensus state to the non-consensus ones. At the same time, the transition from a non-consensus state to the consensus state occurs with the rate 1 (up to the time-scale parameter *β*). Intuitively, in the model with one selected consensus state the probability of transition to a non-consensus state should be smaller than the probability of transition to the consensus state; thus, *λ *should be less than 1. Indeed, if *λ *≥ 1, then *π*_*c *_≤ 1/4, *π*_*n *_≥ 1/4 and we have three equiprobable consensus states.

If the consensus is strong, then *π*_*c *_≥ 1/2, and, consequently, 0 <*λ *≤ 1/3. Note that we can not impose any condition on *μ *other than *μ *> 0.

### Conservation and transition of nucleotides

The goal of this section is to compare the transitional probabilities between different states in our model. We will see that the relations we get have clear biological interpretation.

#### Simple cases

Let a unique consensus exist in our model, i.e. 0 <*λ *< 1. It is not difficult to obtain the following relations between the transitional probabilities, which hold for any *λ *∈ (0, 1), *u *> 0.

1. **P**_*cc*_(*u*) > **P**_*cn*_(*u*);

2. **P**_*cc*_(*u*) > **P**_*nc*_(*u*);

3. **P**_*cc*_(*u*) > **P**_*nm*_(*u*) for any *μ *> 0;

4. **P**_*nn*_(*u*) > **P**_*cn*_(*u*) for any positive *λ *and *μ*;

5. **P**_*nc*_(*u*) > **P**_*cn*_(*u*);

6. **P**_*nn*_(*u*) > **P**_*nm*_(*u*) for any *μ *> 0.

**Figure 1:**
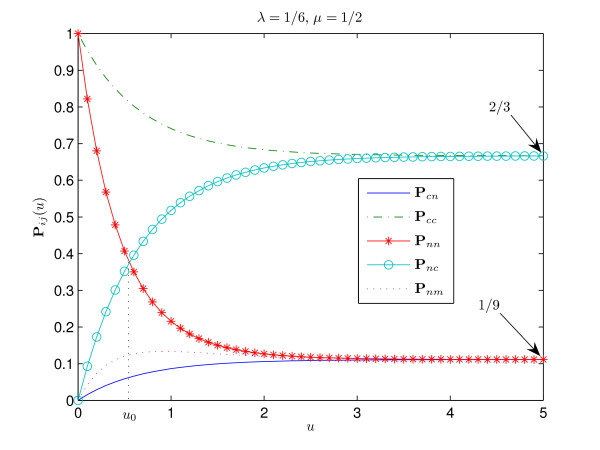
**Transitional probabilities P**_*cn*_, **P**_*cc*_, **P**_*nn*_, **P**_*nc*_, **P**_*nm *_**for ***λ *= 1/6, ***μ ***= 1/2. This figure describes the case **P**_*cc*_(*u*) > **P**_*nn*_(*u*) for all *u *> 0. The consensus is strong, since *π*_*c *_= 2/3. Since the rate of transition between non-consensus states *μ *is greater than the rate of transition from the consensus state *λ*, we have **P**_*cn*_(*u*) <**P**_*nm*_(*u*). The relation between the probability of conservation of a non-consensus nucleotide and the probability of transition from the non-consensus state to the consensus one is shown, **P**_*nn*_(*u*) > **P**_*nc*_(*u*) for *u *∈ (0, *u*_0_) and, vice versa, **P**_*nn*_(*u*) <**P**_*nc*_(*u*) for *u *> *u*_0_. This figure exemplifies all simple cases that hold for any *λ *∈ (0, 1), *μ *> 0, *u *> 0 (see the corresponding paragraph in the main text).

**Figure 2:**
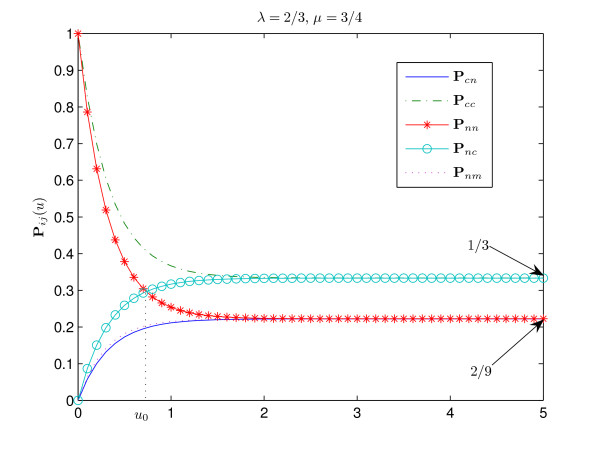
**Transitional probabilities P**_*cn*_, **P**_*cc*_, **P**_*nn*_, **P**_*nc*_, **P**_*nm *_**for ***λ *= 2/3, ***μ ***= 3/4. Here we have **P**_*nn*_(*u*) > **P**_*nc*_(*u*) for *u *∈ (0, *u*_0_) and, vice versa, **P**_*nn*_(*u*) <**P**_*nc*_(*u*) for *u *> *u*_0_. In this figure **P**_*cc*_(*u*) > **P**_*nn*_(*u*) for all *u *> 0, as 3*λ *≤ 2*μ *+ 1. The consensus is weak, since *π*_*c *_= 1/3. Since *μ *> *λ*, we have **P**_*cn*_(*u*) <**P**_*nm*_(*u*). This figure shows all simple cases that hold for any *λ *∈ (0, 1), *μ *> 0, *u *> 0.

**Figure 3:**
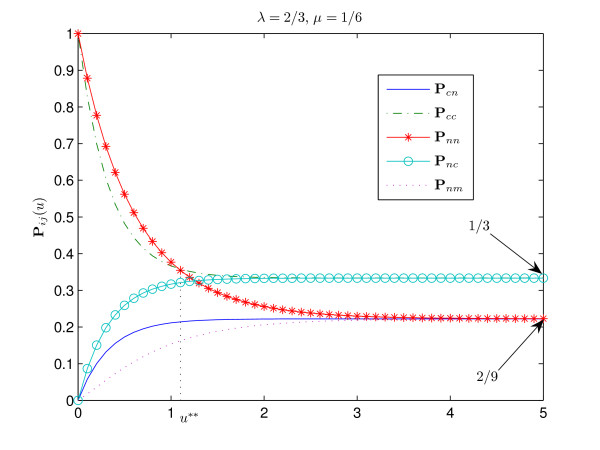
**Transitional probabilities P**_*cn*_, **P**_*cc*_, **P**_*nn*_, **P**_*nc*_, **P**_*nm *_**for ***λ *= 2/3, ***μ ***= 1/6. In this case the probability of conservation of the consensus nucleotide is less than the probability of conservation of a non-consensus nucleotide, **P**_*cc*_(*u*) <**P**_*nn*_(*u*) for *u *∈ (0, u**), since 3*λ *> 2*μ *+ 1 and *λ *> *μ*. The stationary distribution of the consensus state is *π*_*c *_= 1/3. Thus, the consensus is weak. Since *μ *<*λ*, we have **P**_*cn*_(*u*) > **P**_*nm*_(*u*) for all *u *> 0.

**Figure 4:**
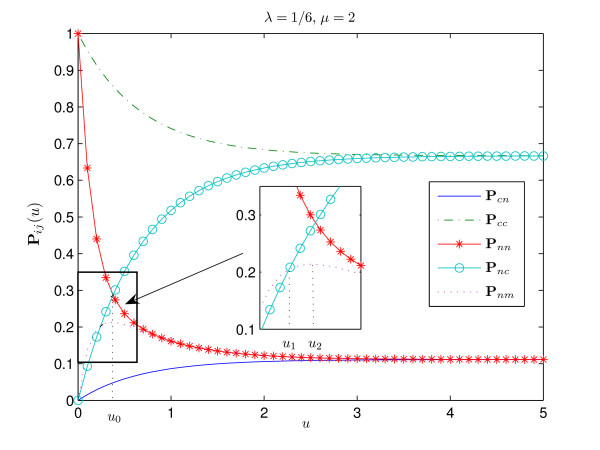
**Transitional probabilities P**_*cn*_, **P**_*cc*_, **P**_*nn*_, **P**_*nc*_, **P**_*nm *_**for ***λ *= 1/6, ***μ ***= 2. Here we have **P**_*cc*_(*u*) > **P**_*nn*_(*u*) for all *u *> 0, since 3*λ *≤ 2*μ *+1. The consensus is strong and the stationary distribution of the consensus state is *π*_*c *_= 2/3. Since *μ *> *λ*, we have **P**_*cn*_(*u*) <**P**_*nm*_(*u*). The probability of substitutions between non-consensus nucleotides **P**_*nm*_(*u*) is not monotone with a maximum point at *u*_2 _= 2 log 7/11 ≈ 0.354. Since *μ *> 1, transitions between non-consensus nucleotides occur more frequently than transitions from a non-consensus to the consensus nucleotide, **P**_*nc*_(*u*) <**P**_*nm*_(*u*) on the time interval *u *∈ (0, *u*_1_), whereas **P**_*nc*_(*u*) > **P**_*nm*_(*u*) for *u *> *u*_1_. This figure also shows the relation between **P**_*nn*_(*u*) and **P**_*nc*_(*u*).

The interpretation of these results is straightforward. Figures 1, 2, 3, 4 show the graphs of the transitional probabilities for different values of *λ *and *μ*. The relations 1–6 between the transitional probabilities are clearly shown in the figures. The first three inequalities show that the probability of conservation of the consensus state is always higher than the probability of transition between the states of different type. Inequalities 1, 4, and 5 imply that the probability of transition from the consensus state to a non-consensus one is always less than the probability of conservation of a nucleotide or the probability of transition from a non-consensus state to the consensus one. Inequalities 3 and 6 show that the probability of transition between two different non-consensus states is always less than the probability of state conservation. All these results correlate well with our intuitive ideas about an evolutionary model with selective pressure. Note that relations 1–6 hold for any positive *μ*, *λ *∈ (0, 1) and for any time period.

#### Interesting cases

The most interesting case is the conservation of the consensus or a non-consensus nucleotide. Recall that **P**_*cc*_(*h*) = 1 - 3 *λβ h *+ *o*(1), **P**_*nn*_(*h*) = 1 - (2*μ *+ 1)*β h *+ *o*(1) for *h *→ 0. Thus, the relation between the probabilities of conservation of the consensus state and conservation of a non-consensus state during the time *u *depends on the relation between 3*λ *and 2*μ *+ 1. We obtained the following result.

1. If 3*λ *≤ 2*μ *+ 1, then

**P**_*cc*_(*u*) > **P**_*nn*_(*u*) for *u *> 0.

2. If 3*λ *> 2*μ *+ 1, *λ *> *μ*, then

Pcc(u)<Pnn(u)for u∈(0,u∗∗),Pcc(u)>Pnn(u)for u>u∗∗.
 MathType@MTEF@5@5@+=feaafiart1ev1aaatCvAUfKttLearuWrP9MDH5MBPbIqV92AaeXatLxBI9gBaebbnrfifHhDYfgasaacH8akY=wiFfYdH8Gipec8Eeeu0xXdbba9frFj0=OqFfea0dXdd9vqai=hGuQ8kuc9pgc9s8qqaq=dirpe0xb9q8qiLsFr0=vr0=vr0dc8meaabaqaciaacaGaaeqabaqabeGadaaakeaafaqaaeGacaaabaacbeGae8huaa1aaSbaaSqaaiabdogaJjabdogaJbqabaGccqGGOaakcqWG1bqDcqGGPaqkcqGH8aapcqWFqbaudaWgaaWcbaGaemOBa4MaemOBa4gabeaakiabcIcaOiabdwha1jabcMcaPaqaaiabbAgaMjabb+gaVjabbkhaYjabbccaGiabdwha1jabgIGiolabcIcaOiabicdaWiabcYcaSiabdwha1naaCaaaleqabaGaey4fIOIaey4fIOcaaOGaeiykaKIaeiilaWcabaGae8huaa1aaSbaaSqaaiabdogaJjabdogaJbqabaGccqGGOaakcqWG1bqDcqGGPaqkcqGH+aGpcqWFqbaudaWgaaWcbaGaemOBa4MaemOBa4gabeaakiabcIcaOiabdwha1jabcMcaPaqaaiabbAgaMjabb+gaVjabbkhaYjabbccaGiabdwha1jabg6da+iabdwha1naaCaaaleqabaGaey4fIOIaey4fIOcaaOGaeiOla4caaaaa@673B@

Here u** = u**(*λ*, *μ*) > 0 is a time moment depending on *λ *and *μ*. More precisely, u** is a non-zero solution of the equation **P**_*cc*_(*u*) = **P**_*nn*_(*u*). Let *F *(*u*) = **P**_*cc*_(*u*) - **P**_*nn*_(*u*). It can be shown that for *λ *> (2*μ *+ 1)/3 and *λ *> *μ*

u∗∗>u∗=13(λ−μ)log⁡[9λ−12(3μ+1)]>0.
 MathType@MTEF@5@5@+=feaafiart1ev1aaatCvAUfKttLearuWrP9MDH5MBPbIqV92AaeXatLxBI9gBaebbnrfifHhDYfgasaacH8akY=wiFfYdH8Gipec8Eeeu0xXdbba9frFj0=OqFfea0dXdd9vqai=hGuQ8kuc9pgc9s8qqaq=dirpe0xb9q8qiLsFr0=vr0=vr0dc8meaabaqaciaacaGaaeqabaqabeGadaaakeaacqWG1bqDdaahaaWcbeqaaiabgEHiQiabgEHiQaaakiabg6da+iabdwha1naaCaaaleqabaGaey4fIOcaaOGaeyypa0ZaaSaaaeaatCvAUfeBSjuyZL2yd9gzLbvyNv2CaeHbuLwBLnhiov2DGi1BTfMBaGabaiaa=fdaaeaacaWFZaGaa8hkaGGaciab+T7aSjabgkHiTiab+X7aTjabcMcaPaaacyGGSbaBcqGGVbWBcqGGNbWzdaWadaqaamaalaaabaGaeGyoaKJae43UdWMaeyOeI0IaeGymaedabaGaeGOmaiJaeiikaGIaeG4mamJae4hVd0Maey4kaSIaeGymaeJaeiykaKcaaaGaay5waiaaw2faaiabg6da+iabicdaWiabc6caUaaa@5DFD@

Indeed,

F(u)=1−λ3λ+1+9λ−13(3λ+1)e−(3λ+1)u−23e−(3μ+1)u,
 MathType@MTEF@5@5@+=feaafiart1ev1aaatCvAUfKttLearuWrP9MDH5MBPbIqV92AaeXatLxBI9gBaebbnrfifHhDYfgasaacH8akY=wiFfYdH8Gipec8Eeeu0xXdbba9frFj0=OqFfea0dXdd9vqai=hGuQ8kuc9pgc9s8qqaq=dirpe0xb9q8qiLsFr0=vr0=vr0dc8meaabaqaciaacaGaaeqabaqabeGadaaakeaacqWGgbGrcqGGOaakcqWG1bqDcqGGPaqkcqGH9aqpdaWcaaqaaiabigdaXiabgkHiTGGaciab=T7aSbqaaiabiodaZiab=T7aSjabgUcaRiabigdaXaaacqGHRaWkdaWcaaqaaiabiMda5iab=T7aSjabgkHiTiabigdaXaqaaiabiodaZiabcIcaOiabiodaZiab=T7aSjabgUcaRiabigdaXiabcMcaPaaacqWGLbqzdaahaaWcbeqaaiabgkHiTiabcIcaOiabiodaZiab=T7aSjabgUcaRiabigdaXiabcMcaPiabdwha1baakiabgkHiTmaalaaabaGaeGOmaidabaGaeG4mamdaaiabdwgaLnaaCaaaleqabaGaeyOeI0IaeiikaGIaeG4mamJae8hVd0Maey4kaSIaeGymaeJaeiykaKIaemyDauhaaOGaeiilaWcaaa@5E89@

and *F'*(*u*) = 0 if and only if

2(3*μ *+ 1) *e*^-(3*μ*+1)*u *^= (9*λ *- 1)*e*^-(3*λ*+1)*u*^.

The solution of the latter equation is u*. If 3*λ *> 2*μ *+ 1 and *λ *> *μ*, then u* > 0 and *F *increases for *u *> u* and decreases for *u *<u*. At the same time, *F*(0) = 0. Thus, in this case *F*(*u*) < 0 for *u *∈ (0, u**) and *F*(*u*) > 0 for *u *> u**. This implies the second statement.

Further, if 3*λ *≤ 2*μ *+ 1 and *λ *> *μ*, then u* < 0 and *F'*(*u*) > 0 for all *u *> 0. Therefore, *F*(*u*) increases for *u *> 0 and *F*(*u*) > 0, since *F*(0) = 0. Next, for *λ *= *μ *we obtain that

*F*(*u*) ≥ 3(1 - *λ*)(1 - *e*^-(3*λ*+1)*u*^) > 0

for any *u *> 0. Thus, *F*(*u*) > 0 if 3*λ *≤ 2*μ *+ 1, and the first inequality follows.

The second relation partly describes the case of the weak consensus (Fig. 3). Since 3*λ *+ 1 > 2(*μ *+ 1), the stationary distribution of the consensus state is estimated from above as

πc=13λ+1<12(μ+1)<12.
 MathType@MTEF@5@5@+=feaafiart1ev1aaatCvAUfKttLearuWrP9MDH5MBPbIqV92AaeXatLxBI9gBaebbnrfifHhDYfgasaacH8akY=wiFfYdH8Gipec8Eeeu0xXdbba9frFj0=OqFfea0dXdd9vqai=hGuQ8kuc9pgc9s8qqaq=dirpe0xb9q8qiLsFr0=vr0=vr0dc8meaabaqaciaacaGaaeqabaqabeGadaaakeaaiiGacqWFapaCdaWgaaWcbaGaem4yamgabeaakiabg2da9maalaaabaGaeGymaedabaGaeG4mamJae83UdWMaey4kaSIaeGymaedaaiabgYda8maalaaabaGaeGymaedabaGaeGOmaiJaeiikaGIae8hVd0Maey4kaSIaeGymaeJaeiykaKcaaiabgYda8maalaaabaGaeGymaedabaGaeGOmaidaaiabc6caUaaa@4275@

At the same time, the first relation holds both in the case of strong consensus *π*_*c*_≥ 1/2 (Fig. 1) and in the case of weak consensus 1/4 <*π*_*c *_< 1/2 (Fig. 2).

The second interesting result is a relation between the probability of conservation of the non-consensus nucleotide and the probability of transition from a non-consensus state to the consensus state. We have

Pnn(u)>Pnc(u),u∈(0,u0),Pnn(u)<Pnc(u),u>u0,
 MathType@MTEF@5@5@+=feaafiart1ev1aaatCvAUfKttLearuWrP9MDH5MBPbIqV92AaeXatLxBI9gBaebbnrfifHhDYfgasaacH8akY=wiFfYdH8Gipec8Eeeu0xXdbba9frFj0=OqFfea0dXdd9vqai=hGuQ8kuc9pgc9s8qqaq=dirpe0xb9q8qiLsFr0=vr0=vr0dc8meaabaqaciaacaGaaeqabaqabeGadaaakeaafaqaaeGacaaabaacbeGae8huaa1aaSbaaSqaaiabd6gaUjabd6gaUbqabaGccqGGOaakcqWG1bqDcqGGPaqkcqGH+aGpcqWFqbaudaWgaaWcbaGaemOBa4Maem4yamgabeaakiabcIcaOiabdwha1jabcMcaPiabcYcaSaqaaiabdwha1jabgIGiolabcIcaOiabicdaWiabcYcaSiabdwha1naaBaaaleaacqaIWaamaeqaaOGaeiykaKIaeiilaWcabaGae8huaa1aaSbaaSqaaiabd6gaUjabd6gaUbqabaGccqGGOaakcqWG1bqDcqGGPaqkcqGH8aapcqWFqbaudaWgaaWcbaGaemOBa4Maem4yamgabeaakiabcIcaOiabdwha1jabcMcaPiabcYcaSaqaaiabdwha1jabg6da+iabdwha1naaBaaaleaacqaIWaamaeqaaOGaeiilaWcaaaaa@5D6D@

where *u*_0 _≡ *u*_0_(*λ*, *μ*) is the solution of equation **P**_*nn*_(*u*) = **P**_*nc*_(*u*). It can be shown that *u*_0 _is always positive. This relation shows that the probability of conservation of a non-consensus nucleotide is less than the probability of transition to the consensus nucleotide from a non-consensus one for sufficiently long time *u *> *u*_0_. However, on the interval (0, *u*_0_) the opposite inequality holds (see Fig. 1, 2).

#### Less interesting cases

It remains to study the relations between **P**_*nm *_and **P**_*cn*_, **P**_*nc*_. From the practical point of view, these cases are not very interesting, since the events of transition between different non-consensus states can be hardly observed on practice. However, we consider them for the sake of completeness.

The following relations imply that the transitional probability from the consensus to a non-consensus state could be less or greater than the transitional probability between two different non-consensus states depending on the relation between *μ *and *λ *(see Fig. 2 and 3):

Pcn(u)<Pnm(u),μ>λ,Pcn(u)=Pnm(u),μ=λ,Pcn(u)>Pnm(u),μ<λ,
 MathType@MTEF@5@5@+=feaafiart1ev1aaatCvAUfKttLearuWrP9MDH5MBPbIqV92AaeXatLxBI9gBaebbnrfifHhDYfgasaacH8akY=wiFfYdH8Gipec8Eeeu0xXdbba9frFj0=OqFfea0dXdd9vqai=hGuQ8kuc9pgc9s8qqaq=dirpe0xb9q8qiLsFr0=vr0=vr0dc8meaabaqaciaacaGaaeqabaqabeGadaaakeaafaqaaeWacaaabaacbeGae8huaa1aaSbaaSqaaiabdogaJjabd6gaUbqabaGccqGGOaakcqWG1bqDcqGGPaqkcqGH8aapcqWFqbaudaWgaaWcbaGaemOBa4MaemyBa0gabeaakiabcIcaOiabdwha1jabcMcaPiabcYcaSaqaaGGaciab+X7aTjabg6da+iab+T7aSjabcYcaSaqaaiab=bfaqnaaBaaaleaacqWGJbWycqWGUbGBaeqaaOGaeiikaGIaemyDauNaeiykaKIaeyypa0Jae8huaa1aaSbaaSqaaiabd6gaUjabd2gaTbqabaGccqGGOaakcqWG1bqDcqGGPaqkcqGGSaalaeaacqGF8oqBcqGH9aqpcqGF7oaBcqGGSaalaeaacqWFqbaudaWgaaWcbaGaem4yamMaemOBa4gabeaakiabcIcaOiabdwha1jabcMcaPiabg6da+iab=bfaqnaaBaaaleaacqWGUbGBcqWGTbqBaeqaaOGaeiikaGIaemyDauNaeiykaKIaeiilaWcabaGae4hVd0MaeyipaWJae43UdWMaeiilaWcaaaaa@6DC6@

Indeed, if *μ *> *λ*, then *δ *> *α *and the transition rate *d *between the non-consensus states is greater than the transition rate from the consensus state to non-consensus. Clearly, in this case **P**_*nm *_has to be greater than **P**_*cn *_(see Fig. 2 and Fig. 3).

The next case concerns the relation **P**_*nm *_and **P**_*nc *_for *λ *∈ (0, 1) (see Fig. 1 and 4):

Pnc(u)>Pnm(u),μ∈(0,1),u>0Pnc(u)<Pnm(u),μ≥1,u∈(0,u1)Pnc(u)>Pnm(u),μ≥1,u>u1,
 MathType@MTEF@5@5@+=feaafiart1ev1aaatCvAUfKttLearuWrP9MDH5MBPbIqV92AaeXatLxBI9gBaebbnrfifHhDYfgasaacH8akY=wiFfYdH8Gipec8Eeeu0xXdbba9frFj0=OqFfea0dXdd9vqai=hGuQ8kuc9pgc9s8qqaq=dirpe0xb9q8qiLsFr0=vr0=vr0dc8meaabaqaciaacaGaaeqabaqabeGadaaakeaafaqaaeWadaaabaacbeGae8huaa1aaSbaaSqaaiabd6gaUjabdogaJbqabaGccqGGOaakcqWG1bqDcqGGPaqkcqGH+aGpcqWFqbaudaWgaaWcbaGaemOBa4MaemyBa0gabeaakiabcIcaOiabdwha1jabcMcaPiabcYcaSaqaaGGaciab+X7aTjabgIGiolabcIcaOiabicdaWiabcYcaSiabigdaXiabcMcaPiabcYcaSaqaaiabdwha1jabg6da+iabicdaWaqaaiab=bfaqnaaBaaaleaacqWGUbGBcqWGJbWyaeqaaOGaeiikaGIaemyDauNaeiykaKIaeyipaWJae8huaa1aaSbaaSqaaiabd6gaUjabd2gaTbqabaGccqGGOaakcqWG1bqDcqGGPaqkcqGGSaalaeaacqGF8oqBcqGHLjYScqaIXaqmcqGGSaalaeaacqWG1bqDcqGHiiIZcqGGOaakcqaIWaamcqGGSaalcqWG1bqDdaWgaaWcbaGaeGymaedabeaakiabcMcaPaqaaiab=bfaqnaaBaaaleaacqWGUbGBcqWGJbWyaeqaaOGaeiikaGIaemyDauNaeiykaKIaeyOpa4Jae8huaa1aaSbaaSqaaiabd6gaUjabd2gaTbqabaGccqGGOaakcqWG1bqDcqGGPaqkcqGGSaalaeaacqGF8oqBcqGHLjYScqaIXaqmcqGGSaalaeaacqWG1bqDcqGH+aGpcqWG1bqDdaWgaaWcbaGaeGymaedabeaakiabcYcaSaaaaaa@837D@

where *u*_1 _= *u*_1_(*λ*, *μ*) is a solution of the equation **P**_*nc*_(*u*) = **P**_*nm*_(*u*).

An interesting fact is that the probability **P**_*nm*_(*u*) is not monotone in *u *for *μ *> *λ *(see Fig. 4). If *μ *> *λ*, then **P**_*nm *_increases on (0, *u*_2_) and decreases for *u *> *u*_2_, where

u2=13(μ−λ)log⁡(3μ+1).
 MathType@MTEF@5@5@+=feaafiart1ev1aaatCvAUfKttLearuWrP9MDH5MBPbIqV92AaeXatLxBI9gBaebbnrfifHhDYfgasaacH8akY=wiFfYdH8Gipec8Eeeu0xXdbba9frFj0=OqFfea0dXdd9vqai=hGuQ8kuc9pgc9s8qqaq=dirpe0xb9q8qiLsFr0=vr0=vr0dc8meaabaqaciaacaGaaeqabaqabeGadaaakeaacqWG1bqDdaWgaaWcbaGaeGOmaidabeaakiabg2da9maalaaabaGaeGymaedabaGaeG4mamJaeiikaGccciGae8hVd0MaeyOeI0Iae83UdWMaeiykaKcaaiGbcYgaSjabc+gaVjabcEgaNjabcIcaOiabiodaZiab=X7aTjabgUcaRiabigdaXiabcMcaPiabc6caUaaa@4377@

**Figure 5:**
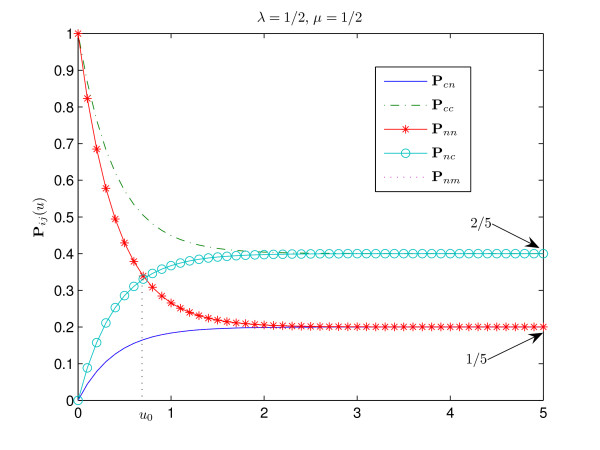
**Transitional probabilities for ***λ *= *μ *= 1/2. This case is similar to the one of Fig. 1, since here 3*λ *≤ 2*μ *+ 1 and, consequently, the probability of consensus conservation is always greater than the probability of conservation of a non-consensus nucleotide, **P**_*cc*_(*u*) > **P**_*nn*_(*u*) for all *u *> 0. As *λ *= *μ*, we have **P**_*nm *_= **P**_*cn*_.

**Figure 6:**
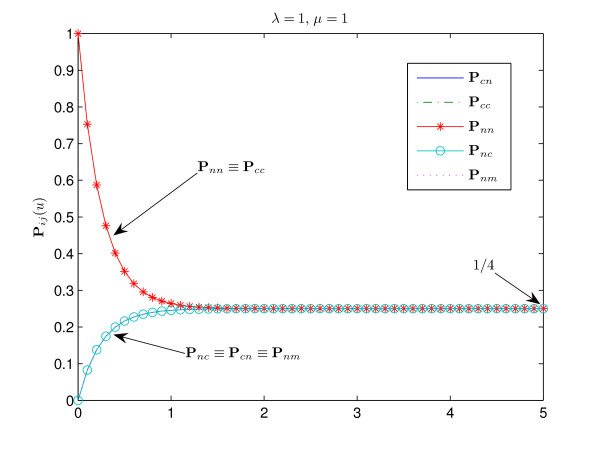
**Degenerate case ***λ *= *μ *= 1. In this case all states are equiprobable, the stationary distribution of the process *π *= (1/4, 1/4, 1/4, 1/4) and **P**_*nn *_≡ **P**_*cc*_, **P**_*nc *_≡ **P**_*cn *_≡ **P**_*nm*_.

Finally, consider the case *μ *= *λ*. As it has been shown above, **P**_*cc*_(*u*) > **P**_*nn*_(*u*) for *u *> 0 (see Fig. 5). It is easy to see that in this case **P**_*nm *_≡ **P**_*cn*_. The function **P**_*nm*_(*u*) is monotonically increasing for all *u*. Note that in the degenerate case *λ *= *μ *= 1 all states are equiprobable, the stationary distribution of the process *π *= (1/4, 1/4, 1/4, 1/4) and **P**_*nn *_= **P**_*cc*_, **P**_*nc *_= **P**_*cn *_= **P**_*nm *_(see Fig. 6).

### Generalization

The obtained results can be generalized on the following model. Consider a Markov chain X˜
 MathType@MTEF@5@5@+=feaafiart1ev1aaatCvAUfKttLearuWrP9MDH5MBPbIqV92AaeXatLxBI9gBaebbnrfifHhDYfgasaacH8akY=wiFfYdH8Gipec8Eeeu0xXdbba9frFj0=OqFfea0dXdd9vqai=hGuQ8kuc9pgc9s8qqaq=dirpe0xb9q8qiLsFr0=vr0=vr0dc8meaabaqaciaacaGaaeqabaqabeGadaaakeaacuWGybawgaacaaaa@2DF4@(*t*) with *M *+ *N *states {*g*_1_, ..., *g*_*M*_, *g*_*M*+1_, ..., *g*_*M*+*N *_}, where the states *g*_1_, ..., *g*_*M *_are consensus states, and the states *g*_*M*+1_, ..., *g*_*M*+*N *_are non-consensus ones. Define the transition rate matrix A˜=(q˜ij)
 MathType@MTEF@5@5@+=feaafiart1ev1aaatCvAUfKttLearuWrP9MDH5MBPbIqV92AaeXatLxBI9gBaebbnrfifHhDYfgasaacH8akY=wiFfYdH8Gipec8Eeeu0xXdbba9frFj0=OqFfea0dXdd9vqai=hGuQ8kuc9pgc9s8qqaq=dirpe0xb9q8qiLsFr0=vr0=vr0dc8meaabaqaciaacaGaaeqabaqabeGadaaakeaacuWGbbqqgaacaiabg2da9iabcIcaOiqbdghaXzaaiaWaaSbaaSqaaiabdMgaPjabdQgaQbqabaGccqGGPaqkaaa@34E6@ for this process by

q˜ij={α,i,j=1,...,M,i≠jγ,i=1,...,M,j=M+1,...,M+Nβ,i=M+1,...,M+N,j=1,...,Mδ,i,j=M+1,...,M+N,i≠j,q˜ii={−(M−1)α−Nγ,i=1,...,M−Mβ−(N−1)δ,i=M+1,...,M+N,
 MathType@MTEF@5@5@+=feaafiart1ev1aaatCvAUfKttLearuWrP9MDH5MBPbIqV92AaeXatLxBI9gBaebbnrfifHhDYfgasaacH8akY=wiFfYdH8Gipec8Eeeu0xXdbba9frFj0=OqFfea0dXdd9vqai=hGuQ8kuc9pgc9s8qqaq=dirpe0xb9q8qiLsFr0=vr0=vr0dc8meaabaqaciaacaGaaeqabaqabeGadaaakeaafaqaaeGabaaabaGafmyCaeNbaGaadaWgaaWcbaGaemyAaKMaemOAaOgabeaakiabg2da9maaceaabaqbaeaabqWaaaaabaacciGae8xSdeMaeiilaWcabaGaemyAaKMaeiilaWIaemOAaOMaeyypa0JaeGymaeJaeiilaWIaeiOla4IaeiOla4IaeiOla4IaeiilaWIaemyta0KaeiilaWcabaGaemyAaKMaeyiyIKRaemOAaOgabaGae83SdCMaeiilaWcabaGaemyAaKMaeyypa0JaeGymaeJaeiilaWIaeiOla4IaeiOla4IaeiOla4IaeiilaWIaemyta0KaeiilaWcabaGaemOAaOMaeyypa0Jaemyta0Kaey4kaSIaeGymaeJaeiilaWIaeiOla4IaeiOla4IaeiOla4IaeiilaWIaemyta0Kaey4kaSIaemOta4eabaGae8NSdiMaeiilaWcabaGaemyAaKMaeyypa0Jaemyta0Kaey4kaSIaeGymaeJaeiilaWIaeiOla4IaeiOla4IaeiOla4IaeiilaWIaemyta0Kaey4kaSIaemOta4KaeiilaWcabaGaemOAaOMaeyypa0JaeGymaeJaeiilaWIaeiOla4IaeiOla4IaeiOla4IaeiilaWIaemyta0eabaGae8hTdqMaeiilaWcabaGaemyAaKMaeiilaWIaemOAaOMaeyypa0Jaemyta0Kaey4kaSIaeGymaeJaeiilaWIaeiOla4IaeiOla4IaeiOla4IaeiilaWIaemyta0Kaey4kaSIaemOta4KaeiilaWcabaGaemyAaKMaeyiyIKRaemOAaOMaeiilaWcaaaGaay5EaaaabaGafmyCaeNbaGaadaWgaaWcbaGaemyAaKMaemyAaKgabeaakiabg2da9maaceaabaqbaeaabiGaaaqaaiabgkHiTiabcIcaOiabd2eanjabgkHiTiabigdaXiabcMcaPiab=f7aHjabgkHiTiabd6eaojab=n7aNjabcYcaSaqaaiabdMgaPjabg2da9iabigdaXiabcYcaSiabc6caUiabc6caUiabc6caUiabcYcaSiabd2eanbqaaiabgkHiTiabd2eanjab=j7aIjabgkHiTiabcIcaOiabd6eaojabgkHiTiabigdaXiabcMcaPiab=r7aKjabcYcaSaqaaiabdMgaPjabg2da9iabd2eanjabgUcaRiabigdaXiabcYcaSiabc6caUiabc6caUiabc6caUiabcYcaSiabd2eanjabgUcaRiabd6eaojabcYcaSaaaaiaawUhaaaaaaaa@C579@

where *α*, *β*, *γ*, *δ*, are positive unknown parameters.

In this case we have two groups of *M *consensus and *N *non-consensus states. We use the same notation for subscripts of transitional probabilities P˜ij
 MathType@MTEF@5@5@+=feaafiart1ev1aaatCvAUfKttLearuWrP9MDH5MBPbIqV92AaeXatLxBI9gBaebbnrfifHhDYfgasaacH8akY=wiFfYdH8Gipec8Eeeu0xXdbba9frFj0=OqFfea0dXdd9vqai=hGuQ8kuc9pgc9s8qqaq=dirpe0xb9q8qiLsFr0=vr0=vr0dc8meaabaqaciaacaGaaeqabaqabeGadaaakeaaieqacuWFqbaugaacamaaBaaaleaacqWGPbqAcqWGQbGAaeqaaaaa@30CE@. If *c *and *d *denote different consensus states, *n *and *m *stand for different non-consensus states as before, we have the following transitional probabilities:

Pcc(t)=ββM+γN+γNM(βM+γN)e−(βM+γN)t+M−1Me−(αM+γN)t,Pcd(t)=ββM+γN+γNM(βM+γN)e−(βM+γN)t−1Me−(αM+γN)t,Pnc(t)=ββM+γN−ββM+γNe−(βM+γN)t,Pcn(t)=γβM+γN−γβM+γNe−(βM+γN)t,Pnn(t)=γβM+γN+βΜN(βM+γN)e−(βM+γN)t+N−1Ne−(βM+δN)t,Pnm(t)=γβM+γN+βNN(βM+γN)e−(βM+γN)t−1Ne−(βM+δN)t.
 MathType@MTEF@5@5@+=feaafiart1ev1aaatCvAUfKttLearuWrP9MDH5MBPbIqV92AaeXatLxBI9gBaebbnrfifHhDYfgasaacH8akY=wiFfYdH8Gipec8Eeeu0xXdbba9frFj0=OqFfea0dXdd9vqai=hGuQ8kuc9pgc9s8qqaq=dirpe0xb9q8qiLsFr0=vr0=vr0dc8meaabaqaciaacaGaaeqabaqabeGadaaakeaafaqaaeGbbaaaaeaaieqacqWFqbaudaWgaaWcbaGaem4yamMaem4yamgabeaakiabcIcaOiabdsha0jabcMcaPiabg2da9maalaaabaacciGae4NSdigabaGae4NSdiMaemyta0Kaey4kaSIae43SdCMaemOta4eaaiabgUcaRmaalaaabaGae43SdCMaemOta4eabaGaemyta0KaeiikaGIae4NSdiMaemyta0Kaey4kaSIae43SdCMaemOta4KaeiykaKcaaiabdwgaLnaaCaaaleqabaGaeyOeI0IaeiikaGIae4NSdiMaemyta0Kaey4kaSIae43SdCMaemOta4KaeiykaKIaemiDaqhaaOGaey4kaSYaaSaaaeaacqWGnbqtcqGHsislcqaIXaqmaeaacqWGnbqtaaGaemyzau2aaWbaaSqabeaacqGHsislcqGGOaakcqGFXoqycqWGnbqtcqGHRaWkcqGFZoWzcqWGobGtcqGGPaqkcqWG0baDaaGccqGGSaalaeaacqWFqbaudaWgaaWcbaGaem4yamMaemizaqgabeaakiabcIcaOiabdsha0jabcMcaPiabg2da9maalaaabaGae4NSdigabaGae4NSdiMaemyta0Kaey4kaSIae43SdCMaemOta4eaaiabgUcaRmaalaaabaGae43SdCMaemOta4eabaGaemyta0KaeiikaGIae4NSdiMaemyta0Kaey4kaSIae43SdCMaemOta4KaeiykaKcaaiabdwgaLnaaCaaaleqabaGaeyOeI0IaeiikaGIae4NSdiMaemyta0Kaey4kaSIae43SdCMaemOta4KaeiykaKIaemiDaqhaaOGaeyOeI0YaaSaaaeaacqaIXaqmaeaacqWGnbqtaaGaemyzau2aaWbaaSqabeaacqGHsislcqGGOaakcqGFXoqycqWGnbqtcqGHRaWkcqGFZoWzcqWGobGtcqGGPaqkcqWG0baDaaGccqGGSaalaeaacqWFqbaudaWgaaWcbaGaemOBa4Maem4yamgabeaakiabcIcaOiabdsha0jabcMcaPiabg2da9maalaaabaGae4NSdigabaGae4NSdiMaemyta0Kaey4kaSIae43SdCMaemOta4eaaiabgkHiTmaalaaabaGae4NSdigabaGae4NSdiMaemyta0Kaey4kaSIae43SdCMaemOta4eaaiabdwgaLnaaCaaaleqabaGaeyOeI0IaeiikaGIae4NSdiMaemyta0Kaey4kaSIae43SdCMaemOta4KaeiykaKIaemiDaqhaaOGaeiilaWcabaGae8huaa1aaSbaaSqaaiabdogaJjabd6gaUbqabaGccqGGOaakcqWG0baDcqGGPaqkcqGH9aqpdaWcaaqaaiab+n7aNbqaaiab+j7aIjabd2eanjabgUcaRiab+n7aNjabd6eaobaacqGHsisldaWcaaqaaiab+n7aNbqaaiab+j7aIjabd2eanjabgUcaRiab+n7aNjabd6eaobaacqWGLbqzdaahaaWcbeqaaiabgkHiTiabcIcaOiab+j7aIjabd2eanjabgUcaRiab+n7aNjabd6eaojabcMcaPiabdsha0baakiabcYcaSaqaaiab=bfaqnaaBaaaleaacqWGUbGBcqWGUbGBaeqaaOGaeiikaGIaemiDaqNaeiykaKIaeyypa0ZaaSaaaeaacqGFZoWzaeaacqGFYoGycqWGnbqtcqGHRaWkcqGFZoWzcqWGobGtaaGaey4kaSYaaSaaaeaacqGFYoGycqGFCoqtaeaacqWGobGtcqGGOaakcqGFYoGycqWGnbqtcqGHRaWkcqGFZoWzcqWGobGtcqGGPaqkaaGaemyzau2aaWbaaSqabeaacqGHsislcqGGOaakcqGFYoGycqWGnbqtcqGHRaWkcqGFZoWzcqWGobGtcqGGPaqkcqWG0baDaaGccqGHRaWkdaWcaaqaaiabd6eaojabgkHiTiabigdaXaqaaiabd6eaobaacqWGLbqzdaahaaWcbeqaaiabgkHiTiabcIcaOiab+j7aIjabd2eanjabgUcaRiab+r7aKjabd6eaojabcMcaPiabdsha0baakiabcYcaSaqaaiab=bfaqnaaBaaaleaacqWGUbGBcqWGTbqBaeqaaOGaeiikaGIaemiDaqNaeiykaKIaeyypa0ZaaSaaaeaacqGFZoWzaeaacqGFYoGycqWGnbqtcqGHRaWkcqGFZoWzcqWGobGtaaGaey4kaSYaaSaaaeaacqGFYoGycqWGobGtaeaacqWGobGtcqGGOaakcqGFYoGycqWGnbqtcqGHRaWkcqGFZoWzcqWGobGtcqGGPaqkaaGaemyzau2aaWbaaSqabeaacqGHsislcqGGOaakcqGFYoGycqWGnbqtcqGHRaWkcqGFZoWzcqWGobGtcqGGPaqkcqWG0baDaaGccqGHsisldaWcaaqaaiabigdaXaqaaiabd6eaobaacqWGLbqzdaahaaWcbeqaaiabgkHiTiabcIcaOiab+j7aIjabd2eanjabgUcaRiab+r7aKjabd6eaojabcMcaPiabdsha0baakiabc6caUaaaaaa@623B@

Thus, this process has the stationary distribution π˜
 MathType@MTEF@5@5@+=feaafiart1ev1aaatCvAUfKttLearuWrP9MDH5MBPbIqV92AaeXatLxBI9gBaebbnrfifHhDYfgasaacH8akY=wiFfYdH8Gipec8Eeeu0xXdbba9frFj0=OqFfea0dXdd9vqai=hGuQ8kuc9pgc9s8qqaq=dirpe0xb9q8qiLsFr0=vr0=vr0dc8meaabaqaciaacaGaaeqabaqabeGadaaakeaaiiGacuWFapaCgaacaaaa@2E7F@ = (*π*_*c*_, ..., *π*_*c*_, *π*_*n*_, ..., *π*_*n*_) with

πc=ββM+γN,πn=γβM+γN.
 MathType@MTEF@5@5@+=feaafiart1ev1aaatCvAUfKttLearuWrP9MDH5MBPbIqV92AaeXatLxBI9gBaebbnrfifHhDYfgasaacH8akY=wiFfYdH8Gipec8Eeeu0xXdbba9frFj0=OqFfea0dXdd9vqai=hGuQ8kuc9pgc9s8qqaq=dirpe0xb9q8qiLsFr0=vr0=vr0dc8meaabaqaciaacaGaaeqabaqabeGadaaakeaafaqabeqacaaabaacciGae8hWda3aaSbaaSqaaiabdogaJbqabaGccqGH9aqpdaWcaaqaaiab=j7aIbqaaiab=j7aIjabd2eanjabgUcaRiab=n7aNjabd6eaobaacqGGSaalaeaacqWFapaCdaWgaaWcbaGaemOBa4gabeaakiabg2da9maalaaabaGae83SdCgabaGae8NSdiMaemyta0Kaey4kaSIae83SdCMaemOta4eaaaaacqGGUaGlaaa@4753@

Clearly, *β *> *γ*, since the first *M *states are the consensus ones. Next, compare the probabilities of conservation of the consensus and non-consensus states **P**_*cc *_and **P**_*nn*_. In a similar way, we obtain that there exists t* > 0 such that **P**_*cc*_(*t*) <**P**_*nn*_(*t*) for *t *∈ (0, t*). Analyzing the probabilities **P**_*cc *_and **P**_*nn *_we can show that this is possible only for *δ *(*N *- 1) - *α *(*M *- 1) + *β M *- *γ N *< 0. Then the frequency of the consensus state (stationary distribution of consensus) is estimated from above as

πc=ββM+γN<β2βM+δ(N−1)−α(M−1).
 MathType@MTEF@5@5@+=feaafiart1ev1aaatCvAUfKttLearuWrP9MDH5MBPbIqV92AaeXatLxBI9gBaebbnrfifHhDYfgasaacH8akY=wiFfYdH8Gipec8Eeeu0xXdbba9frFj0=OqFfea0dXdd9vqai=hGuQ8kuc9pgc9s8qqaq=dirpe0xb9q8qiLsFr0=vr0=vr0dc8meaabaqaciaacaGaaeqabaqabeGadaaakeaaiiGacqWFapaCdaWgaaWcbaGaem4yamgabeaakiabg2da9maalaaabaGae8NSdigabaGae8NSdiMaemyta0Kaey4kaSIae83SdCMaemOta4eaaiabgYda8maalaaabaGaeqOSdigabaGaeGOmaiJae8NSdiMaemyta0Kaey4kaSIae8hTdqMaeiikaGIaemOta4KaeyOeI0IaeGymaeJaeiykaKIaeyOeI0Iae8xSdeMaeiikaGIaemyta0KaeyOeI0IaeGymaeJaeiykaKcaaiabc6caUaaa@4EC8@

If *M *= 1, *N *= 3, then

πc<β2β+2δ<12.
 MathType@MTEF@5@5@+=feaafiart1ev1aaatCvAUfKttLearuWrP9MDH5MBPbIqV92AaeXatLxBI9gBaebbnrfifHhDYfgasaacH8akY=wiFfYdH8Gipec8Eeeu0xXdbba9frFj0=OqFfea0dXdd9vqai=hGuQ8kuc9pgc9s8qqaq=dirpe0xb9q8qiLsFr0=vr0=vr0dc8meaabaqaciaacaGaaeqabaqabeGadaaakeaaiiGacqWFapaCdaWgaaWcbaGaem4yamgabeaakiabgYda8maalaaabaGae8NSdigabaGaeGOmaiJae8NSdiMaey4kaSIaeGOmaiJae8hTdqgaaiabgYda8maalaaabaGaeGymaedabaGaeGOmaidaaiabc6caUaaa@3C81@

This condition coincides with the condition on weak consensus obtained for the case of four states with one consensus (*M *= 1, *N *= 3) considered above.

### Comparison with the Molecular Evolution Theory

In the framework of the molecular evolution theory, the element *a*_*ij *_of the transition rate matrix is considered to be proportional to the product of the mutation rate *p*_*ij *_and the probability of fixation of a mutation *f*_*ij*_, *a*_*ij *_= *kp*_*ij*_*f*_*ij*_, where *k *is an arbitrary scaling constant [47]. As in [45] we start from the simplest Jukes–Cantor (1 - *p*)-scheme, to which we introduce selection. Thus, we ignore the difference between transitions and transversions in *p*_*ij*_.

TFBS regulating different genes in the same genome most likely evolve independently and thus the nucleotide composition *π*_*i *_at the respective positions of different TFBS occurrences approximates the equilibrium frequencies. With these equilibrium frequencies at hand it is possible to relate *p*_*ij *_and *f*_*ij *_with the equation (see [47])

fij∝log⁡(πjpjiπipij)1−πipijπjpji.
 MathType@MTEF@5@5@+=feaafiart1ev1aaatCvAUfKttLearuWrP9MDH5MBPbIqV92AaeXatLxBI9gBaebbnrfifHhDYfgasaacH8akY=wiFfYdH8Gipec8Eeeu0xXdbba9frFj0=OqFfea0dXdd9vqai=hGuQ8kuc9pgc9s8qqaq=dirpe0xb9q8qiLsFr0=vr0=vr0dc8meaabaqaciaacaGaaeqabaqabeGadaaakeaacqWGMbGzdaWgaaWcbaGaemyAaKMaemOAaOgabeaakiabg2Hi1oaalaaabaGagiiBaWMaei4Ba8Maei4zaC2aaeWaaeaadaWcaaqaaGGaciab=b8aWnaaBaaaleaacqWGQbGAaeqaaOGaemiCaa3aaSbaaSqaaiabdQgaQjabdMgaPbqabaaakeaacqWFapaCdaWgaaWcbaGaemyAaKgabeaakiabdchaWnaaBaaaleaacqWGPbqAcqWGQbGAaeqaaaaaaOGaayjkaiaawMcaaaqaaiabigdaXiabgkHiTmaalaaabaGae8hWda3aaSbaaSqaaiabdMgaPbqabaGccqWGWbaCdaWgaaWcbaGaemyAaKMaemOAaOgabeaaaOqaaiab=b8aWnaaBaaaleaacqWGQbGAaeqaaOGaemiCaa3aaSbaaSqaaiabdQgaQjabdMgaPbqabaaaaaaakiabc6caUaaa@5997@

In our case, for the substitution rate between three non-consensus positions we obtain *π*_*i *_= *π*_*j *_= *π*_*n *_and *p*_*ij *_= *p*_*ji *_= *p*_*nm*_, which yields *f*_*nm*_∝ 1 by the l'Hôpital rule as in [47]. Thus, *δ *= *r*_*nm *_= *k*_*pnm*_.

For the substitutions between non-consensus and consensus positions, *r*_*nc*_, both the selection preferences and mutation asymmetry come into consideration. In this case the "asymmetry constant" *λ *is crucial, which satisfies the inequality *r*_*cn*_/*r*_*nc *_= *p*_*n*_/*p*_*c *_= *λ *< 1. The following expression is valid:

fcn∝log⁡(λpncpcn)1−λ−1pcnpnc.
 MathType@MTEF@5@5@+=feaafiart1ev1aaatCvAUfKttLearuWrP9MDH5MBPbIqV92AaeXatLxBI9gBaebbnrfifHhDYfgasaacH8akY=wiFfYdH8Gipec8Eeeu0xXdbba9frFj0=OqFfea0dXdd9vqai=hGuQ8kuc9pgc9s8qqaq=dirpe0xb9q8qiLsFr0=vr0=vr0dc8meaabaqaciaacaGaaeqabaqabeGadaaakeaacqWGMbGzdaWgaaWcbaGaem4yamMaemOBa4gabeaakiabg2Hi1oaalaaabaGagiiBaWMaei4Ba8Maei4zaC2aaeWaaeaaiiGacqWF7oaBdaWcaaqaaiabdchaWnaaBaaaleaacqWGUbGBcqWGJbWyaeqaaaGcbaGaemiCaa3aaSbaaSqaaiabdogaJjabd6gaUbqabaaaaaGccaGLOaGaayzkaaaabaGaeGymaeJaeyOeI0Iae83UdW2aaWbaaSqabeaacqGHsislcqaIXaqmaaGcdaWcaaqaaiabdchaWnaaBaaaleaacqWGJbWycqWGUbGBaeqaaaGcbaGaemiCaa3aaSbaaSqaaiabd6gaUjabdogaJbqabaaaaaaakiabc6caUaaa@51CD@

This fixation rate is linked with the Kimura selection constant [48]* s *by the relation *f*_*cn *_= (1 - *e*^-2*s*^)/(1 - *e*^-2*Ns*^), where *N *is the population size.

If the mutation rate is symmetric, *p*_*nc *_= *p*_*cn*_, then *f*_*cn *_∝ *λ *log(1/*λ*)/(1 - *λ*). Conversely, for the non-consensus to consensus substitution *f*_*nc*_∝ log(1/*λ*)/(1 - *λ*) = *f*_*cn*_/*λ*, the greater flux from a non-consensus state to the consensus maintains a greater consensus frequency. Note that in alignments of sites in a single genome we can observe only the equilibrium constants *π*_*n*_, *π*_*c *_(which actually are rather rough approximations), thus the assumption *p*_*nc *_= *p*_*cn *_may be too strong, and the above general formula for *f*_*cn *_might be more relevant.

The coefficients in the matrix *A *for the symmetric mutation rates are given by

α=rcn=kpcnλlog⁡(1/λ)1−λ,β=rnc=kpnclog⁡(1/λ)1−λ.
 MathType@MTEF@5@5@+=feaafiart1ev1aaatCvAUfKttLearuWrP9MDH5MBPbIqV92AaeXatLxBI9gBaebbnrfifHhDYfgasaacH8akY=wiFfYdH8Gipec8Eeeu0xXdbba9frFj0=OqFfea0dXdd9vqai=hGuQ8kuc9pgc9s8qqaq=dirpe0xb9q8qiLsFr0=vr0=vr0dc8meaabaqaciaacaGaaeqabaqabeGadaaakeaafaqaaeGabaaabaacciGae8xSdeMaeyypa0JaemOCai3aaSbaaSqaaiabdogaJjabd6gaUbqabaGccqGH9aqpcqWGRbWAcqWGWbaCdaWgaaWcbaGaem4yamMaemOBa4gabeaakmaalaaabaGae83UdWMagiiBaWMaei4Ba8Maei4zaCMaeiikaGIaeGymaeJaei4la8Iae83UdWMaeiykaKcabaGaeGymaeJaeyOeI0Iae83UdWgaaiabcYcaSaqaaiab=j7aIjabg2da9iabdkhaYnaaBaaaleaacqWGUbGBcqWGJbWyaeqaaOGaeyypa0Jaem4AaSMaemiCaa3aaSbaaSqaaiabd6gaUjabdogaJbqabaGcdaWcaaqaaiGbcYgaSjabc+gaVjabcEgaNjabcIcaOiabigdaXiabc+caViab=T7aSjabcMcaPaqaaiabigdaXiabgkHiTiab=T7aSbaacqGGUaGlaaaaaa@6579@

If the background mutation rate is identical for all consensus and non-consensus nucleotides, we obtain *p*_*cn *_= *p*_*nc *_= *p*_*nm *_= *p *and *p *may be merged with the constant *k*. In this most simple case we obtain

α=kλlog⁡(1/λ)1−λ,β=klog⁡(1/λ)1−λ,δ=k;
 MathType@MTEF@5@5@+=feaafiart1ev1aaatCvAUfKttLearuWrP9MDH5MBPbIqV92AaeXatLxBI9gBaebbnrfifHhDYfgasaacH8akY=wiFfYdH8Gipec8Eeeu0xXdbba9frFj0=OqFfea0dXdd9vqai=hGuQ8kuc9pgc9s8qqaq=dirpe0xb9q8qiLsFr0=vr0=vr0dc8meaabaqaciaacaGaaeqabaqabeGadaaakeaafaqabeqadaaabaacciGae8xSdeMaeyypa0Jaem4AaS2aaSaaaeaacqWF7oaBcyGGSbaBcqGGVbWBcqGGNbWzcqGGOaakcqaIXaqmcqGGVaWlcqWF7oaBcqGGPaqkaeaacqaIXaqmcqGHsislcqWF7oaBaaGaeiilaWcabaGae8NSdiMaeyypa0Jaem4AaS2aaSaaaeaacyGGSbaBcqGGVbWBcqGGNbWzcqGGOaakcqaIXaqmcqGGVaWlcqWF7oaBcqGGPaqkaeaacqaIXaqmcqGHsislcqWF7oaBaaGaeiilaWcabaGae8hTdqMaeyypa0Jaem4AaSMaei4oaSdaaaaa@571B@

and, consequently, *β *> *δ *> *α *. This is the simplest generalization of the Jukes–Cantor model for the case with introduced selection.

It should be noted that in this case *μ *= *δ*/*β *= (1 - *λ*)/log(1/*λ*), which implies *μ *> *λ*. Thus, 3*λ *≤ 2*μ *+ 1 and we are in Case 1 of "Interesting Cases" for which **P**_*cc*_(*u*) > **P**_*nn*_(*u*) for *u *> 0.

### Conservation of non-consensus nucleotides at the reduced mutation rate

Previously [8] we have observed that non-consensus nucleotides may be highly conserved in the alignments of orthologous TFBS in bacterial genomes. On the other hand, as shown above, the non-consensus nucleotide in the alignment of orthologous sites from different species cannot be more conserved than the consensus nucleotide if we adopt the Kimura model with the identical mutation rate for all pairs.

One way to explain the observation made in [8] is to drop the equivalence of non-consensus nucleotides and assume that different non-consensus nucleotides are under different selection in the sites regulating different rows of orthologous genes. Interestingly, it is possible to observe such conservation pattern in the model with an identical probabilities of mutation and fixation for all non-consensus nucleotides in all orthologous site rows, with a single preferred nucleotide, the consensus, the same in all sites. The only necessary relaxation of the model is to drop the condition *p*_*cn *_= *p*_*nc *_= *p*_*nm *_= *p *for the condition *p*_*nm *_<*p*_*nc *_= *p*_*cn*_. Doing this it is possible to satisfy the inequalities determining Case 2 of "Interesting Cases": *μ *<*λ*, 3*λ *> 2*μ *+ 1.

The condition *p*_*nm *_<*p*_*nc *_= *p*_*cn *_means that the rate of direct mutations from one non-consensus nucleotide to another one is lower than the mutation rate in pairs involving the consensus nucleotide. A possible example of such specific reduction of the mutation rate comes from correlations of nucleotides occupying different positions of the same binding site. Assume that two positions within the site are not independent and must be occupied by correlated nucleotides. These may be, e.g., adjacent positions in a DNA site or base-paired positions in an RNA structure. Assume also that if any of the two positions is occupied by the consensus nucleotide, the correlating nucleotide may be arbitrary. Conversely, if one position is occupied by a non-consensus nucleotide, the other position should be occupied with some specific nucleotide, e.g. the complementary one in the case of an RNA structure.

In this case, the preferred pathway from a non-consensus nucleotide to another non-consensus nucleotide would become not via a direct mutation, but via an intermediate mutation into the consensus nucleotide. For example, if "C" is both cytosine and consensus, and for some case this position is correlated with another one, so that only A-A, T-T, and G-G pairs involving the non-consensus nucleotides at the first position are allowed, then only mutation A>C is valid, whereas mutations A>T and A>G are forbidden, and may occur via mutation into C and then the compensating mutation in the second position of the site. This simple model to some extent agrees with recent studies demonstrating that protein-DNA interactions are rather complex and probably may not be described by a simple position-independent model such as a positional weight matrix [49].

At the same time, this effect is not in contradiction with the uniform distribution of non-consensus nucleotides obtained from alignments of multiple TFBS regulating different genes in the same genome. It also allows for high conservation of some non-consensus nucleotides in the alignment of orthologous transcription factor binding sites from different species. Indeed, if the context-dependent pattern of conservation is specific to a particular position in a particular set of orthologous TFBSs, exactly this type of behavior may be expected.

## Conclusion

The evolutionary model derived here can be generalized to the case of *M *consensus and *N *non-consensus nucleotides that are equiprobable, respectively. Thus, for *M *= 2, *N *= 2 and *α *= *β*, *γ *= *δ*. we get the case of neutral evolution with different rates of transitions and transversions [40]. If *M *= 1, *N *= 3, then we have the evolution model under constant selective pressure considered in this paper.

One somewhat non-obvious feature of this model is the existence of a combination of the rate of transition between non-consensus states *μ *and the rate of transition from the consensus state *λ*, for which the conservation of the consensus nucleotide in a sequence alignment can be lower than the conservation of a neutral (non-consensus) nucleotide. However, this can be observed only for the case of a weak consensus 1/4 <*N*_*c *_< 1/2 and for a relatively short time interval *u *∈ (0, u**), and a mutation matrix with different elements, e.g. context-dependent.

Models of this type can be applied not only for the analysis of regulatory sites, but in other situations, e.g. for the analysis of functional sites in proteins [50] or analysis of evolution in the case of nucleotide biases [41-44,51,52].

In future work, we intend to estimate the parameters of the model from the data based on the method of maximum likelihood trees. Consider a Markov process *X*(*t*) describing the evolution at some fixed position. We assume that we observe only the endpoints of *k *different paths of *X*(*t*) depending on the evolution branch. In other words, our data is a set of *k *nucleotides at a fixed position in *k *genomes. The parameter *λ *can be easily estimated by the 'naive' estimator λ^
 MathType@MTEF@5@5@+=feaafiart1ev1aaatCvAUfKttLearuWrP9MDH5MBPbIqV92AaeXatLxBI9gBaebbnrfifHhDYfgasaacH8akY=wiFfYdH8Gipec8Eeeu0xXdbba9frFj0=OqFfea0dXdd9vqai=hGuQ8kuc9pgc9s8qqaq=dirpe0xb9q8qiLsFr0=vr0=vr0dc8meaabaqaciaacaGaaeqabaqabeGadaaakeaaiiGacuWF7oaBgaqcaaaa@2E77@ = (1/*N*_*c *_- 1)/3, where *N*_*c *_is the frequency of the consensus nucleotide at the fixed position within *k *genomes. This estimator is obtained from the formula for the stationary distribution of the consensus state *π*_*c *_= 1/(3*λ *+ 1). However, it is a good approximation of the true value of *λ*. The preliminary analysis of numerically simulated data shows that the performance of the maximum likelihood estimator is also rather good. On the other hand, it is not clear whether it is possible to construct an explicit estimator for the rate of transition between non-consensus states *μ *from the data, although *μ *participates in the expression for transitional probabilities.

The inspiration for the constructed model was the example of a set of orthologous transcription factors interacting with the cognate regulatory regions. However, it appears that evolution of sequences under a low selection pressure is a more widespread phenomenon. Indeed, a recent study [53] demonstrated that the force causing conservation of some non-coding genome regions of human, mouse and chimpanzee can be explained by a rather small selective pressure at the genomic level. A similar problem appears in the context of the CG composition of genomic regions [54]. Again, in this case the selective pressure appears to be low, although unlike the previous examples, here two nucleotides become selected for rather than one consensus nucleotide. The appropriate model in this case includes differences in the transition and the transversion rates, which makes the model more complicated, and probably would result in more complex time behavior of the substitution probabilities.

Anyhow, the emerging huge amount of data on orthologous non-coding regions, which has became available recently, brings forward a problem of modelling evolution with a selection pressure at finite times.

## Methods

The numerical simulations and figures were produced using Matlab.

## Authors' contributions

MSG and VJM conceived the study. FNE and EAK developed the model. FNE performed numerical simulations. FNE, VJM and MSG wrote the paper. All authors have read and approved the final version.
